# Identification and Characterization of a *Cis* Antisense RNA of the *rpoH* Gene of *Salmonella enterica* Serovar Typhi

**DOI:** 10.3389/fmicb.2018.00978

**Published:** 2018-05-15

**Authors:** Changyan Xiong, Xuejiao Li, Juanli Liu, Xin Zhao, Shungao Xu, Xinxiang Huang

**Affiliations:** ^1^Department of Biochemistry and Molecular Biology, School of Medicine, Jiangsu University, Zhenjiang, China; ^2^Department of Forensic Medicine, School of Medicine, Jiangsu University, Zhenjiang, China; ^3^Department of Laboratory Diagnosis, Changhai Hospital, The Second Military Medical University, Shanghai, China

**Keywords:** *Salmonella enterica* serovar Typhi, antisense RNA, ArpH, *rpoH*, invasion

## Abstract

Antisense RNAs from complementary strands of protein coding genes regulate the expression of genes involved in many cellular processes. Using deep sequencing analysis of the *Salmonella enterica* serovar Typhi (*S.* Typhi) transcriptome, a novel antisense RNA encoded on the strand complementary to the *rpoH* gene was revealed. In this study, the molecular features of this antisense RNA were assessed using northern blotting and rapid amplification of cDNA ends. The 3,508 nt sequence of RNA was identified as the antisense RNA of the *rpoH* gene and was named ArpH. ArpH was found to attenuate the invasion of HeLa cells by *S.* Typhi by regulating the expression of SPI-1 genes. In an *rpoH* mutant strain, the invasive capacity of *S.* Typhi was increased, whereas overexpression of ArpH positively regulates *rpoH* mRNA levels. Results of this study suggest that the *cis*-encoded antisense RNA ArpH is likely to affect the invasive capacity of *S.* Typhi by regulating the expression of *rpoH*.

## Introduction

*Salmonella enterica* serovar Typhi (*S.* Typhi) is an important Gram-negative pathogenic bacterium that can cause symptoms ranging from mild diarrhea to serious systemic infections, such as typhoid fever, owing to many virulence genes (Dougan and Baker, 2014). Concentrated areas of virulence genes, called *Salmonella* pathogenicity islands (SPI), are located on the bacterial chromosome, plasmids, or phages (Matsui et al., 2008). The majority of virulence genes are located on SPIs on the chromosome. Virulence genes protect bacteria from damage caused by the host immune system. However, *S.* Typhi also interacts with the host and changes the host cell environment. The ability of *S.* Typhi to invade and survive in non-phagocytic cells is an indication of its pathogenicity. These pathogenic mechanisms have been linked to SPIs (Nieto et al., 2016).

Pathogens can quickly adapt to changing circumstances by regulating expression of their virulence genes during the process of infection. Many non-coding RNAs (ncRNAs) involved in complex regulatory mechanisms have been identified. Research has demonstrated that ncRNAs are involved in transcriptional and post-transcriptional regulation via the formation of complexes with DNA or proteins through base pairing interactions (Kaikkonen et al., 2011; Ghosal et al., 2013). Previously, ncRNA was considered to be transcriptional noise that was not involved in protein coding; however, a growing number of studies have confirmed that ncRNA can play a role in regulating the expression of many genes in bacteria, including ABC transport system (Miyakoshi et al., 2015), quorum sensing (Bardill and Hammer, 2012), oxidative stress (Gerstle et al., 2012), acid resistance (Aiso et al., 2011), and virulence genes. ncRNAs are classified as either *trans*-encoded or *cis*-encoded based on their position in the genome. *Trans*-encoded ncRNAs are located between protein coding genes and, because they are located at distant genomic locations, are only partially complementary to their target mRNAs and act via incomplete base pairing. *Cis*-encoded ncRNAs are located within protein-coding genes and, because they are located in the same region, are fully complementary to their target mRNAs (Storz et al., 2011).

**FIGURE 1:**
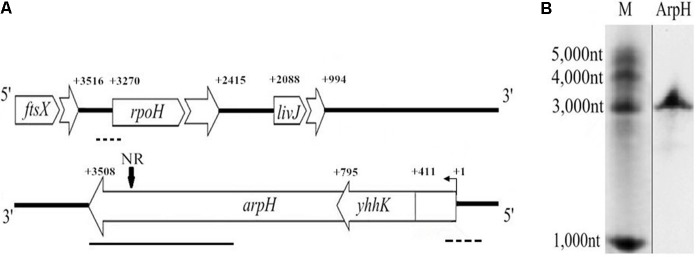
Genomic location and northern blotting analysis of *arpH*. **(A)** Schematic representation of the genomic location of *ftsX*, *rpoH, livJ, yhhK*, and *arpH* genes. NR represents the specific probe used in the northern blot. Dotted line, a deleted fragment in the *rpoH* or *arpH* mutants; bold line, a 1164 bp fragment used for plasmid pBAD-*arpH* construction; bent arrow, transcription start site; +1, experimentally determined transcription start site of *arpH*; +number, gene transcription start or termination site. **(B)** ArpH was detected in *S.* Typhi by northern blotting analysis using an *arpH*-specific digoxigenin-labeled cDNA probe.

We previously conducted deep RNA sequencing analysis of the transcriptome and identified several novel *cis*-encoded antisense RNAs (Dadzie et al., 2013, 2014). A 1164-nt transcript partially encoded by the minus strand of *rpoH* was expressed. The maximum expression of the transcript is from 1887 nt downstream from the start codon of *yhhK* and overlaps the 192-nt region upstream from the *rpoH* gene start codon, as shown by bioinformatic prediction (**Figure 1A**). RpoH activates expression of the *hfq* gene, which encodes an RNA-binding regulatory protein. RpoH also regulates the expression of genes involved in adaptations to environmental stresses, such as thermal stress (Martinez-Salazar et al., 2009; López-Leal et al., 2016). However, the full length and function of the antisense RNA of *rpoH* is not yet known. In the present study, we described the identification and characterization of a novel antisense RNA encoded by the strand complementary to the *rpoH* gene sequence that we named ArpH. We demonstrated that ArpH is likely to affect the invasive capacity of *S.* Typhi by regulating the expression of *rpoH*.

## Materials and Methods

### Bacterial Strains, Plasmids, and Growth Conditions

The strains and plasmids used in this study are listed in **Table 1**. All strains were grown at 37°C in Luria-Bertani (LB) medium supplemented with 100 μg/mL ampicillin or 50 μg/mL kanamycin when required.

**Table 1:** Bacterial strains and plasmids utilized in this study.

	Description	Source
**Strains**		
*S.* Typhi GIFU 10007	Wild type strain; z66^+^	Gifu University
TOP10	*E. coli* host strain	Invitrogen
Δ*arpH*	GIFU10007(Δ*arpH*)	This work
Δ*rpoH*	GIFU10007 (Δ*rpoH*), Kan^r^	This work
WT-pBAD	GIFU10007 carrying pBADMyc-HisA empty plasmid	This work
WT-pBAD-*arpH*	GIFU 10007 carrying pBAD-*arpH*	This work
Δ*rpoH*-pBAD	Δ*rpoH* carrying pBADMyc-HisA empty plasmid	This work
Δ*rpoH*-pBAD-*arpH*	Δ*rpoH* carrying pBAD-*arpH*	This work
*E. coli* DH 5α	*E. coli* host strain of T vector	Invitrogen
Δ*rne*	GIFU10007(Δ*rne*)	Dadzie et al., 2013
Δ*rnc*	GIFU10007(Δ*rnc*)	Dadzie et al., 2013
Δ*rne*-*arpH*	Δ*rne* contained pBAD-*arpH*	This work
Δ*rnc*-*arpH*	Δ*rnc* contained pBAD-*arpH*	This work
**Plasmids**		
pBADMyc-hisA	P_lacO_ promoter; Amp^r^	Invitrogen
pBAD-*arpH*	P_lacO_ promoter, *arpH* insert; Amp^r^	This work
pGEM-T vector	TA clone; Amp^r^	Promega
pET-28a-c(+)	Kan^r^	Laboratory collection
pKD46	Red helper plasmid; Amp^r^	Laboratory collection
pGMB151	Suicide plasmid; *sacB*; Amp^r^	Huang et al., 2004

### Strain Construction

The oligonucleotides used in this study are listed in **Table 2**. To construct the *arpH* mutant strain, primer pairs F1A/F1B and F2A/F2B were used to amplify fragments F1 (467 bp) and F2 (409 bp), which are located 355 nt upstream and 80 nt downstream of the *arpH* gene transcriptional start site, respectively. F1 and F2 were used as templates for a second PCR using primers F1A/F2B to obtain a single 876 bp fragment. *Bam*HI sites were added to the 5′-ends of primers F1A and F2B. The 876 bp fragment was then inserted into the *Bam*HI site of the suicide plasmid pGMB151, carrying the sucrose-sensitivity gene *sacB*. pGMB151 containing the insert was electroporated into the *S.* Typhi wild-type strain. Putative *arpH* mutants were cultured on LB plates containing sucrose. Colonies were screened for inserts by PCR with primers F1A/F2B and by DNA sequencing and mutants were named Δ*arpH* (Huang et al., 2004).

**Table 2:** Oligonucleotides utilized in this study.

Name	Sequence (5′–3′)
**Primers used to construct strains**	
F1A(*Bam* HI)	CTGGGATCCATCGTCACCAACATGCCTTC
F1B	CAAAGCACATGCATAATGTCGGTATTCTGA
F2A	GACATTATGCATGTGCTTTGTGACTCATTA
F2B(*Bam* HI)	ACTGGATCCATATTCCGGCCAGATTTTGC
*rpoH*F1A	AAGAGTGGATGATATTCTCGTTGCTCATCGGCTT TGGCACGGTTGTTGCTCGCTGACGG
*rpoH*F1B	GCCGGATAGCAGCGTAAACGCCTTATCCGGCCT ACAAAAAACAAAACCCCCGAATTCAC
*rpoH*F2A	ACTTTACTCCCGATTG
*rpoH*F2B	ATGGCTCATAACACCC
PA (*Nco*I)	AAGCCATGGTAAGCGAAGCGACATCGG
PB (*Xho* I)	AAGCTCGAGTCCCTGTTGTCTCTTCCC
**Primers and adaptor used for RACE**	
5′RACE RT	TATTATCCGCGCTCGCTGGCTGG
5′RACE outer primer	CATGGCTACATGCTGACAGCCTA
5′RACE inner primer	CGCGGATCCACAGCCTACTGATGATCAGTCGATG
5′RACE GSP1	CCATCCGGCTTTCTTTTA
5′RACE GSP2	CGATCAGTGTACCGAAAC
3′RACE adaptor	Phosphate-UUCACUGUUCUUAGCGGCCGCAUGCUC-idT
3′RACE adaptor primer	GGCCGCTAAGAACAGTGAA
3′RACE GSP1	ACGTATTCGTGAATTTCT
3′RACE GSP2	GCATAGTTACGAGCAATA
**Probes used for northern hybridization**	
ArpH-NR	AGGGCGATCTGGAAGCAGCTAAAACGCTGATC CTGTCTCACCTGCGCTTTGTTG
5s-qF	TTGTCTGGCGGCAGTAGC
5s-qR	TTTGATGCCTGGCAGTTC
**Primers used for real-time PCR**	
*sodB-*FA	CGAATTACCTGCATTACCGT
*sodB*-FB	CAGCGATTTGCCTTCAAACG
*orgA-*FA	TCGCCTGTTGAGGGGATACT
*orgA-*FB	TTTTTTCCATCCACTTCCA
*prgH-*FA	GAACGGCTGTGAGTTTCCAT
*prgH-*FB	GGCGAATCAGGATAAGCAAT
*sipA-*FA	TTGATATGTGCCACCAAAAA
*sipA-*FB	TTTATCTGCAGGAATTTGTG
*invF-*FA	AGGATTAGTGGACACGACATA
*invF-*FB	AAGAAACGCCATAGTCTTCTC

An *rpoH* mutant was constructed using the lambda Red recombinase method (Datsenko and Wanner, 2000). A kanamycin resistance (*kan*) gene was amplified from the pET-28a plasmid using primers *rpoH*F1A/F1B. The *kan* sequences in the amplified fragment were flanked with 50 bp sequences complementary to either end of the *rpoH* promoter region. The deletion fragment is located 171 nt upstream and 60 nt downstream of the *rpoH* gene transcriptional start site. Electrocompetent cells containing the plasmid pKD46 were transformed with the purified PCR product. The transformants were cultured on LB plates containing kanamycin. *rpoH* mutant strains were screened by PCR using the primers *rpoH*F2A/F2B and *rpoH*F2A/F1B and the mutants named Δ*rpoH*.

### Plasmid Construction

To construct the plasmid pBAD-*arpH*, primers PA and PB were used to amplify a 1,182 bp fragment of *arpH*, which consists of 1164bp transcript and 18bp of restriction fragment (**Figure 1A**). The PCR product was cloned into an *Nco*I/*Hind*III-digested pBAD/Myc-His A vector (Invitrogen). The wild-type *S.* Typhi and Δ*rpoH* strains were transformed with the recombinant vector pBAD-*arpH* or the pBAD plasmid alone using electroporation to generate the strains WT-pBAD-*arpH*, WT-pBAD, Δ*rpoH*-pBAD-*arpH*, and Δ*rpoH*-pBAD. RNase E and RNase III mutants were electroporated with pBAD-*arpH* to obtain Δ*rne*-*arpH* and Δ*rnc*-*arpH* (Dadzie et al., 2013).

### RNA Extraction

*Salmonella enterica* serovar Typhi grown overnight in LB broth was diluted 1:100 and grown at 37°C with shaking at 250 rpm. Total RNA was extracted at OD_600_ = 0.8 using the RNeasy Mini kit (Qiagen) according to the manufacturer’s protocol for assessment of the presence of ArpH. For overexpression analysis, *S.* Typhi and Δ*rpoH* carrying pBAD or pBAD-*arpH* plasmids were grown in LB broth until the OD_600_ reached 0.4 and induced by the addition of 0.2% L-arabinose. The cells were harvested by centrifugation at 10,000 × *g* at 4°C. Total RNA was then extracted using TRIzol Reagent (Invitrogen) followed by treatment with RNase-free DNase I (Takara). RNA was quantified using spectrophotometer at 260 nm.

### 5′- and 3′-RACE Analysis

We performed 5′ rapid amplification of cDNA ends (5′-RACE) experiments as previously described (Zhang et al., 2015). A total of 10 units of calf intestine alkaline phosphatase (TakaRa) was added to 5 μg total RNA for 60 min at 50°C. One unit of tobacco acid pyrophosphatase (TakaRa) was then added for 60 min at 37°C. Total RNA was ligated using T4 RNA ligase (TakaRa) for 60 min at 16°C. cDNA was synthesized using M-MLV reverse transcriptase (TakaRa) according to the manufacturer’s instructions with an antisense-specific primer (5′RACE RT). The first round of PCR amplification was performed using a 5′-RACE adaptor-specific primer (5′RACE outer primer) and an *arpH*-specific primer (5′RACE GSP1). A second round of PCR amplification was performed using the primers 5′RACE inner primer and 5′RACE GSP2. The PCR products were extracted, subcloned, and sequenced to confirm the transcription initiation site of the *arpH* sequence.

3′-RACE was carried out using a SMARTer RACE kit (TakaRa) according to the manufacturer’s instructions. Total RNA was treated with calf intestine alkaline phosphatase (TakaRa) and ligated to the 5′-phosphorylated 3′-RACE adaptor (3′RACE adaptor). Reverse transcription was performed using an *arpH*-specific primer (3′RACE RT) and an adaptor-specific primer (3′RACE adaptor primer) complementary to the 3′RACE adaptor. The outer and inner PCR reactions were performed using the 3′-RACE adaptor primer and *arpH*-specific primers (3′RACE GSP1 and 3′RACE GSP2). PCR amplification, cloning, and sequencing were performed as described for 5′-RACE.

### Northern Blotting Analysis

To detect ArpH, an *arpH*-specific cDNA probe was used, which was located from 130 nt to 183 nt downstream of the *rpoH* gene initiation codon on the complementary strand (**Figure 1**). A total of 10–20 μg of total RNA was separated on a 6% polyacrylamide gel containing 7 M urea and electroblotted onto Hybond^TM^ N^+^ membranes (GE Healthcare). The cDNA probes were synthesized using a DIG Northern Starter kit (Roche). Membranes were prehybridized in Rapid-Hyb buffer (GE Healthcare). The digoxigenin-labeled riboprobes were hybridized overnight at 42°C with the prehybridized membranes. Membranes were washed as previously described (Zhang et al., 2015). Riboprobed membranes were exposed to KODAK x-ray film at -70°C.

### Quantitative Real-Time PCR

Equal amounts of RNA were reverse transcribed into cDNA using random primers (Takara). The primer pairs used for quantitative real-time PCR (qRT-PCR) analysis are shown in **Table 2**. Reactions were monitored using a C1000 Thermal Cycler (Bio-Rad) and transcript levels were normalized to 5S ribosomal RNA using the 2^-ΔΔ^*^C^*^t^ method (Bader et al., 2003). Each experiment was performed according to the manufacturers’ protocols in triplicate.

### Growth Curves

Strain growth was determined by measuring the OD_600_ using a BioPhotometer (Eppendorf). WT-pBAD and WT-pBAD-*arpH* strains were grown overnight in LB broth containing 100 mg/mL ampicillin, diluted 1:100, and incubated at 37°C with shaking at 250 rpm with the addition of 0.2% L-arabinose. To monitor the growth rate, cells were cultured in LB to an OD_600_ of 0.4 under acid stress (HCl, pH 4.5), oxidative stress (1 mM H_2_O_2_), or osmotic stress (0.3 M NaCl) conditions. The absorbance was measured at 1 h intervals for 24 h and growth curves were determined from biological triplicates.

### Microarray Analysis

For gene expression profiling experiments, single colonies of WT-pBAD and WT-pBAD-*arpH* grown overnight in LB broth containing 100 mg/mL ampicillin were diluted 1:100 and incubated at 37°C with shaking at 250 rpm with 0.2% L-arabinose. Cells were then cultured in LB to an OD_600_ of 0.4 and treated with 1 mM of H_2_O_2_ for 4 h. Total RNA was extracted using the RNeasy Mini kit (Invitrogen) according to the manufacturer’s protocol. Samples were harvested by centrifugation at 10,000 × *g* at 4°C. Total RNA was isolated using TRIzol Reagent (Invitrogen) followed by treatment with RNase-free DNase I (Takara). A total of 25 μg RNA was used as the template for cDNA synthesis. Genomic microarray analysis of *S.* Typhi was performed as described previously (Sethi et al., 2010; Du et al., 2011). The microarray was analyzed by comparing spot intensities of WT-pBAD and WT-pBAD-*arpH* strains (Zhang et al., 2009).

### Epithelial Cell Invasion Assay

HeLa cells (2 × 10^5^) were seeded into 24-well tissue culture plates containing RPMI 1640 medium (Life Technologies) supplemented with 10% fetal bovine serum and incubated at 37°C in 5% CO_2_ for 16 h. *S.* Typhi cultures were grown to an OD_600_ of 0.4. The bacterial cells were washed thoroughly with phosphate-buffered saline at a multiplicity of infection of 20. The monolayers were either lysed with Triton X-100 to evaluate the level of adherence (*T*_0_) or incubated for a further 3 h in medium containing 100 μg/mL gentamicin to eliminate extracellular bacteria and to assess the level of invasion (*T*_90_). The invasiveness of *S.* Typhi was previously demonstrated by our laboratory (Gong et al., 2015).

### Statistical Analysis

Results are presented as mean ± SD. Significant differences between groups were assessed using Student’s *t*-tests or analysis of variance using SPSS software (SPSS Inc.). Differences were considered significant at *P* < 0.05.

## Results

### Identification and Expression of ArpH

Recently, several new ncRNAs were identified by deep sequencing analysis of the *S.* Typhi genome (Dadzie et al., 2013, 2014; Gong et al., 2015; Zhang et al., 2015). The RNA-seq analysis of *S*. Typhi under stress conditions showed a 1164-nt transcript partially encoded by the minus strand of *rpoH*, extending from 192 nt upstream to 972 nt downstream from the start codon of *rpoH* (**Figure 1A**). In the current study, total RNA was extracted from a wild-type *S.* Typhi strain and hybridized with an *arpH*-specific digoxigenin-labeled cDNA probe to confirm the existence of ArpH. We found that the *arpH* fragment was approximately 3,000 nt (**Figure 1B**). 5′-RACE was then performed to map the 5′-end of the *arpH* sequence, which was found to be 411 nt upstream of the *yhhK* initiation codon (**Figure 1A**). 3′-RACE revealed that the 3′-end of the *arpH* sequence was 238 nt upstream of the *rpoH* initiation codon on the complementary strand. The results of RACE analyses indicated that the total length of the *arpH* sequence is 3508 nt.

**FIGURE 2:**
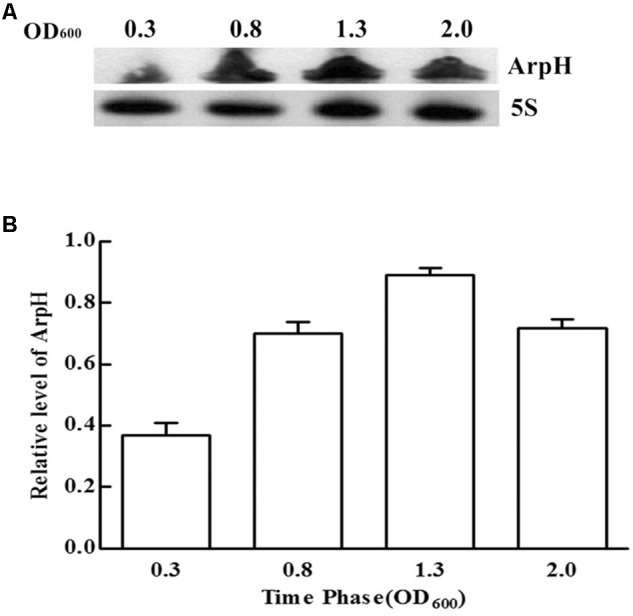
ArpH expression levels in *S.* Typhi grown to different OD_600_ values. **(A)** Total RNA was harvested at an OD_600_ of 0.3, 0.8, 1.3, or 2.0. An *arpH*-probe was used for northern blotting analysis. The 5S rRNA was used as the control. **(B)** The levels of ArpH in *S.* Typhi harvested at an OD_600_ of 0.3, 0.8, 1.3, or 2.0 were measured using qRT-PCR and normalized to 5S rRNA.

**FIGURE 3:**
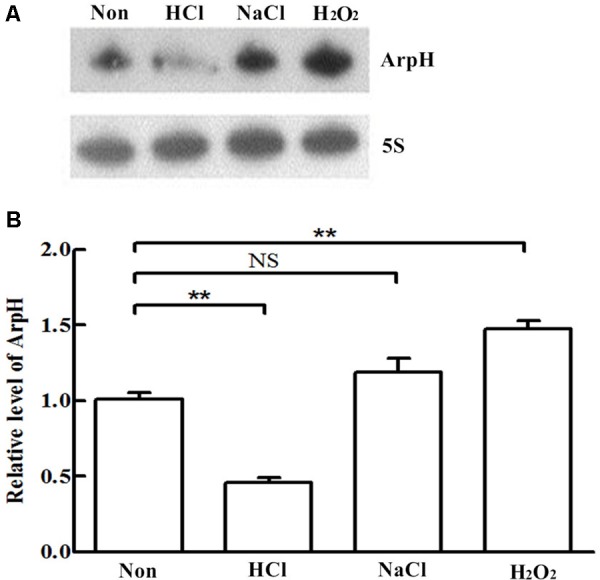
ArpH expression under stress conditions. **(A)** Northern blotting analysis and **(B)** qRT-PCR analysis of total RNA extracted from wild-type *S.* Typhi cultured in LB to an OD_600_ of 0.4 and subjected for 30 min to acid stress (HCl), osmotic stress (NaCl), or oxidative stress (H_2_O_2_). The 5S rRNA was used as the internal reference. ^∗∗^*P* < 0.01. NS, not statistically significant.

We monitored the levels of ArpH over time and under different stress conditions using northern blotting analysis and qRT-PCR. Total RNA was harvested from wild-type *S.* Typhi grown to an OD_600_ of 0.3, 0.8, 1.3, or 2.0, representing the lag phase through the stationary phase (**Figure 2A**). The expression of ArpH was also measured by qRT-PCR (**Figure 2B**). To examine levels of ArpH under stress conditions, we simulated the environment that *Salmonella* encounters upon invasion or within macrophages. Total RNA was extracted after cells were subjected to acid, osmotic, or oxidative stress. Northern blotting and qRT-PCR demonstrated that ArpH levels were lower when *S.* Typhi was exposed to acidic conditions and higher when exposed to oxidative conditions compared to *S.* Typhi grown under normal conditions (**Figures 3A,B**). These results indicate that ArpH is expressed in *S.* Typhi and that its expression changes in response to stress conditions.

### Effect of ArpH Overexpression on *rpoH* mRNA Levels

**FIGURE 4:**
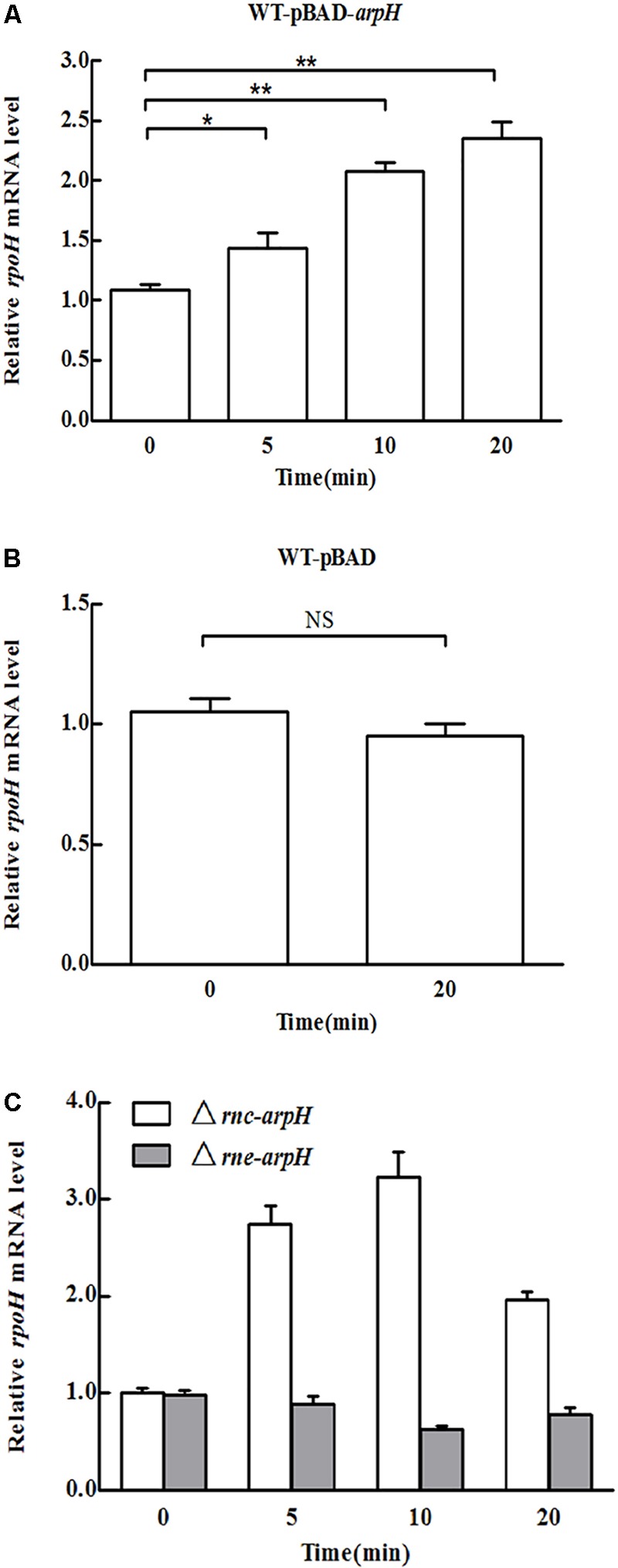
Overexpression of ArpH. mRNA levels of *rpoH* in **(A)** WT-pBAD-*arpH*, **(B)** WT-pBAD, and **(C)** Δ*rnc-arpH* and Δ*rne*-*arpH* strains were measured using qRT-PCR. Total RNA was harvested from strains grown to an OD_600_ of 0.4 after 0, 5, 10, or 20 min of induction with 0.2% L-arabinose. ^∗^*P* < 0.05, ^∗∗^*P* < 0.01. NS, not statistically significant.

To examine the effect of ArpH on its putative mRNA target *rpoH*, we investigated expression levels of *rpoH* mRNA using qRT-PCR after overexpressing the partial *arpH* sequence from the arabinose-inducible recombinant plasmid WT-pBAD-*arpH*. The partial overexpression of the *arpH* did not affect the expression of the *yhhK, livJ*, and *rpoH* gene, which were estimated with qRT-PCR (data not shown). The relative level of ArpH in WT-pBAD-*arpH* strain was significantly higher than the WT-pBAD strain by qRT-PCR (data not shown), which were consistent with our expectation. In contrast to WT-pBAD alone, the mRNA level of *rpoH* increased gradually within 20 min of ArpH overexpression (**Figures 4A,B**). These results indicate that ArpH positively regulates *rpoH* mRNA levels.

RNase E and RNase III are the main endoribonucleases that cleave antisense RNA-induced target mRNA (Saramago et al., 2014). RNase E cuts RNA at a number of single-stranded regions, whereas RNase III cleaves double-stranded RNA (Arraiano et al., 2010). To determine the effect of ArpH overexpression on *rpoH* mRNA levels in RNase E and RNase III mutants, we constructed Δ*rne*-*arpH* and Δ*rnc*-*arpH* strains. The mRNA expression of *rpoH* was sixfold higher in RNase III mutants than in the control strains (**Figure 4C**), whereas the levels were relatively similar in RNase E mutants, indicating that RNase III may play a more significant role in the coupled degradation of the ArpH/RpoH duplex.

### Effect of ArpH Overexpression on *S.* Typhi Growth

**FIGURE 5:**
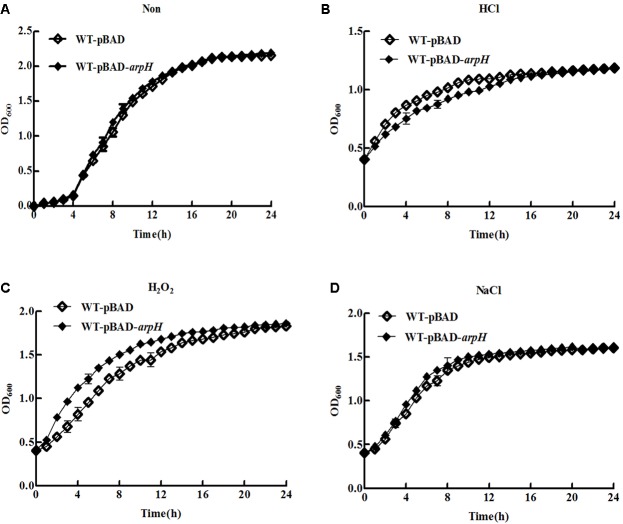
Growth curves of WT-pBAD and WT-pBAD-*arpH* strains. Single colonies of WT-pBAD and WT-pBAD-*arpH* were cultured overnight at 37°C with shaking, diluted 1:100 in LB medium, and induced with 0.2% L-arabinose. **(A)** Growth curves of strains grown under normal conditions were generated by measuring the OD_600_ at 1 h intervals. To assess growth under stress conditions, cells were cultured in LB to an OD_600_ of 0.4 under stress conditions including **(B)** acid stress (HCl, pH 4.5), **(C)** oxidative stress (H_2_O_2_, 1 mM), and **(D)** osmotic stress (NaCl, 0.3 M). Growth curves were generated by measuring the OD_600_ at 1 h intervals.

We investigated the growth of WT-pBAD and WT-pBAD-*arpH* strains over a 24 h period. **Figure 5A** shows that the growth curves of the two strains were similar during the lag and stationary phases; however, during the logarithmic phase, the WT-pBAD-*arpH* strain grew slightly faster than the WT-pBAD strain. Growth curves were also constructed for the WT-pBAD and WT-pBAD-*arpH* strains under different growth conditions. The growth curves for WT-pBAD and WT-pBAD-*arpH* strains were similar under osmotic stress. However, the growth of the WT-pBAD-*arpH* strain was enhanced under oxidative stress during the early logarithmic phase and overexpression of ArpH resulted in significant reduction of bacterial growth under acid stress during the late logarithmic phase (**Figures 5B–D**). Additionally, the growth curves of the WT-pBAD and WT-pBAD-*arpH* strains were similar under thermal stress (data not shown).

### Analysis of Genes Regulated by ArpH

**FIGURE 6:**
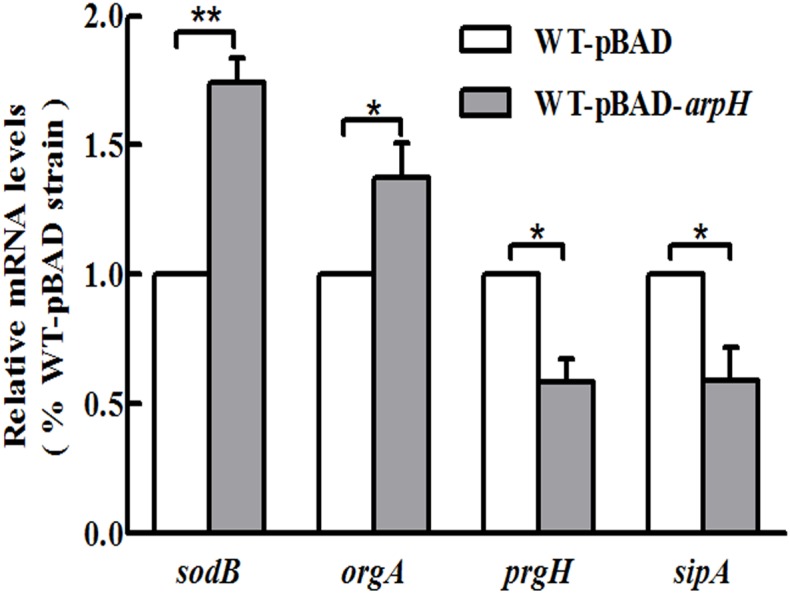
Gene expression levels of *sodB*, *orgA*, *prgH*, and *sipA*. Single colonies of WT-pBAD and WT-pBAD-*arpH* strains were cultured overnight at 37°C with shaking, diluted 1:100 in LB medium, and induced by 0.2% L-arabinose. Cells were cultured in LB to an OD_600_ of 0.4 and treated with 1 mM of H_2_O_2_ for 4 h. Total RNA was harvested and the relative mRNA levels of *sodB*, *orgA*, *prgH*, and *sipA* were measured by qRT-PCR. The 5S rRNA was used as the internal reference. ^∗^*P* < 0.05, ^∗∗^*P* < 0.01.

To investigate the effect of ArpH overexpression in oxidative stress conditions, a WT-pBAD-*arpH* strain was created. The wild-type *S.* Typhi strain was transformed with the recombinant vector pBAD-*arpH* or the pBAD plasmid by electroporation. The gene expression profile of WT-pBAD-*arpH* was assessed using a whole genome microarray to investigate the influence of ArpH overexpression in *S.* Typhi under oxidative stress conditions (Supplementary Material). Microarray results showed differential expression of genes involved in SPI-1 and invasion, flagellar biosynthesis, and virulence between the WT-pBAD-*arpH* and WT-pBAD strains. SPI-1 and invasion-associated genes, including *prgHIK*, *iagA*, *sipCDA*, *invFGA*, *spaKINM*, and *tviACDE*, were downregulated in the WT-pBAD-*arpH* strain. Metabolism-associated genes, including *astB*, *msyB*, *glpD*, *argD*, and *sdhDC*, were also downregulated in the WT-pBAD-*arpH* strain. Expression of flagellum-associated genes, such as *flgBCDEF*, *fliACJHLSZ*, and *flhDC*, were not significantly different in the WT-pBAD-*arpH* strain. However, the superoxide dismutase gene *sodB* and the oxygen-regulated invasion gene *orgA* were upregulated in the WT-pBAD-*arpH* strain. Using qRT-PCR, we confirmed the microarray results by comparing the expression of the genes *sodB*, *orgA*, *prgH*, and *sipA* between the WT-pBAD-*arpH* and WT-pBAD strains (**Figure 6**). These findings suggested that ArpH plays a role in the antioxidant defenses of *S.* Typhi under oxidative stress conditions (Bang et al., 2005).

### *arpH* Deletion Enhances the Invasion of Epithelial Cells by *S.* Typhi

**FIGURE 7:**
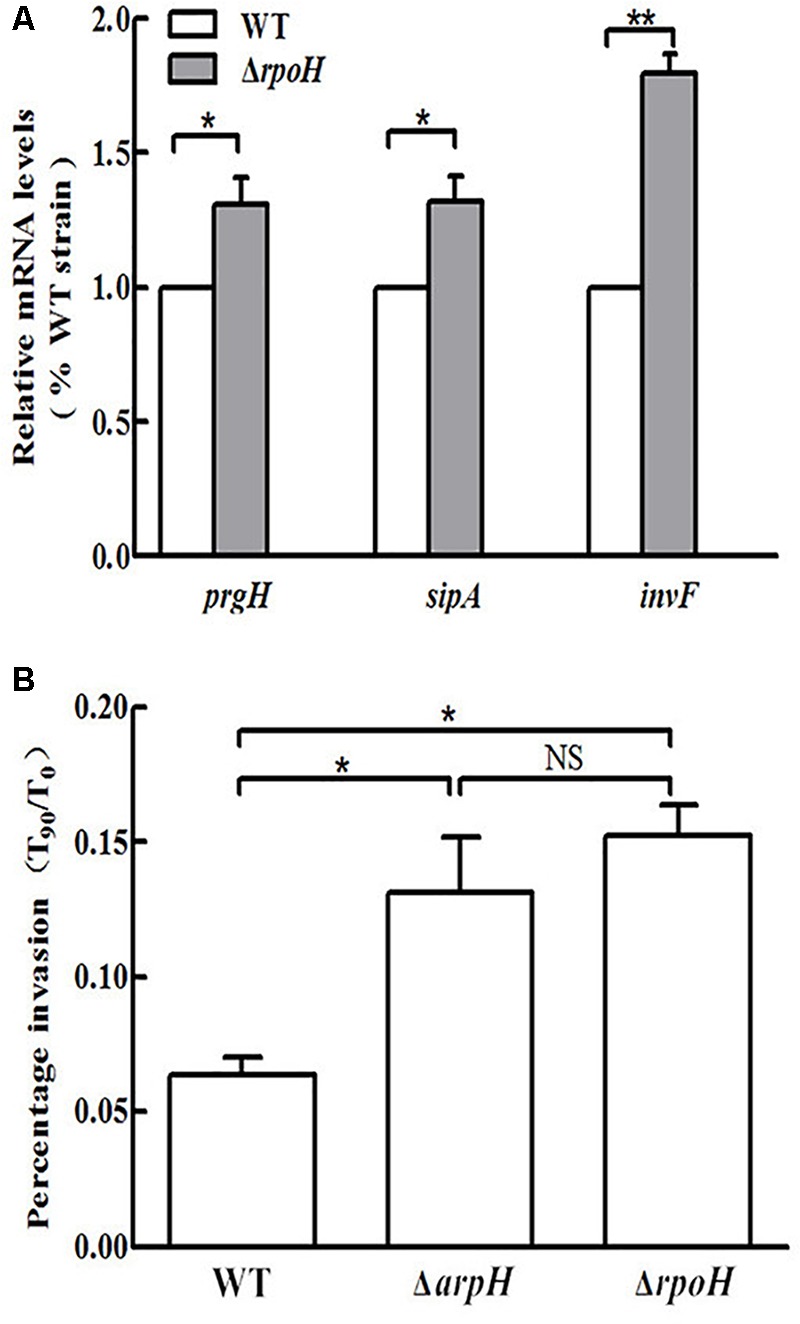
Invasion of HeLa cells by wild-type, Δ*arpH*, and Δ*rpoH* strains. **(A)** Total RNA was harvested and the relative mRNA levels of the invasion-associated genes *prgH*, *sipA*, and *invF* were measured in wild-type (WT) and Δ*rpoH* strains using qRT-PCR. The 5S rRNA was used as the internal reference. **(B)** The invasiveness of *S.* Typhi into HeLa cells was measured by counting the number of bacteria at 0 min (*T*_0_) and after 90 min (*T*_90_). The capacity of *S.* Typhi to invade HeLa cells *in vitro* was determined by the ratio of *T*_90_ to *T*_0_. The data are shown as mean values and standard deviations of three experiments. ^∗^*P* < 0.05, ^∗∗^*P* < 0.01. NS, not statistically significant.

We constructed an *arpH* mutant in which the 435-nt *arpH* fragment was disrupted, whereas the SD box and the ORF structure of *yhhK, livJ*, and *rpoH* were retained intact. The deletion of the *arpH* did not affect the expression of the *yhhK, livJ*, and *rpoH* gene, which were estimated with qRT-PCR (data not shown), and the results were consistent with our expectation. An *rpoH* deletion mutant was also constructed using the lambda Red recombinase method. Because the *arpH*-encoding region overlaps the *rpoH* gene and the *arpH* might influence RpoH function, we investigated the function of *rpoH* in this study. A *rpoH* mutant was constructed, in which the deleted region was only the promoter region of *rpoH*. The deletion of the *rpoH* did not affect the expression of the *arpH* gene, which was estimated with qRT-PCR (data not shown), and found to be consistent with our expectation. To examine the possibility that ArpH may play a role in the invasion of epithelial cells by *Salmonella*, we examined the invasion efficiency of wild-type, Δ*arpH*, and Δ*rpoH* strains into HeLa cells. To confirm these findings, we used qRT-PCR to measure the mRNA levels of the SPI-1 and invasion-associated genes *prgH*, *sipA*, and *invF* (**Figure 7A**). From the results shown in **Figure 7B**, we concluded that the invasion efficiency of the Δ*arpH* strain was significantly higher than that of the wild-type strain. Taken together, these findings indicate that ArpH on RpoH might negatively regulate the invasion of *S.* Typhi into the intestinal epithelium.

### *rpoH* Deletion Upregulates Expression of Invasion-Associated Genes

Transcriptome analysis of the *rpoH* mutant was carried out using a whole genome microarray. Significant upregulation of the invasion-associated genes *prgH*, *sipA*, and *invF* was observed in the Δ*rpoH* strain. In contrast, no significant increase in the expression levels of these genes was observed in the wild-type strain. To confirm the findings of the microarray experiment, we used qRT-PCR to measure the expression of the *prgH*, *sipA*, and *invF* genes (**Figure 7A**). The HeLa cell invasion assay demonstrated that the invasion capacity of the Δ*rpoH* strain was dramatically enhanced compared to the wild-type strain. This result is consistent with previous literature (Matsui et al., 2008). However, the results of the invasion assay demonstrated that there was no significant difference in invasion capacity between the Δ*arpH* and Δ*rpoH* mutants (**Figure 7B**). Taken together, these findings suggest that ArpH might downregulate the invasiveness of *S.* Typhi by affecting the expression of *rpoH*.

### Effect of ArpH Overexpression on Invasiveness of *S.* Typhi

**FIGURE 8:**
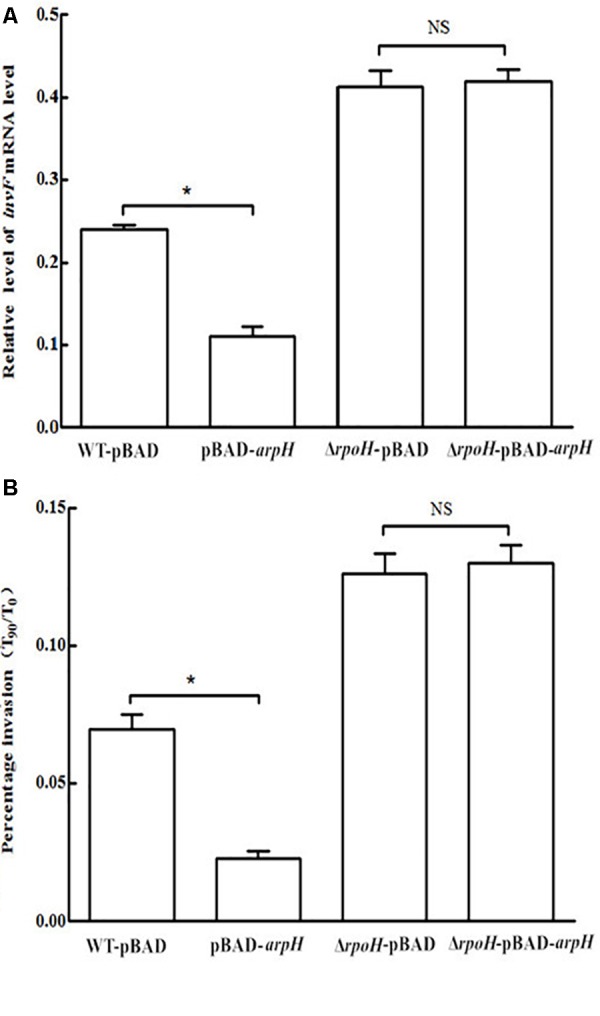
Effect of ArpH overexpression on mRNA levels of *invF* and invasion efficiency of *rpoH* mutants. **(A)** The mRNA levels of the invasion-associated gene *invF* were measured by qRT-PCR. The 5S rRNA was used as the internal reference. **(B)** Comparison of the invasion efficiencies of WT-pBAD-*arpH*, WT-pBAD, Δ*rpoH*-pBAD, and Δ*rpoH*-pBAD-*arpH* into HeLa cells. Invasion of HeLa cells was measured by counting the number of bacteria at 0 min (*T*_0_) and after 90 min (*T*_90_). The capacity of *S.* Typhi to invade HeLa cells *in vitro* was determined by the ratio of *T*_90_ to *T*_0_. The data are shown as mean values and standard deviations of three experiments. ^∗^*P* < 0.05. NS, not statistically significant.

In order to further study the mechanism by which ArpH affects the invasiveness of *S.* Typhi, the wild-type *S.* Typhi and Δ*rpoH* strains were transformed with the recombinant vector pBAD-*arpH* or the pBAD plasmid alone using electroporation to generate the strains WT-pBAD-*arpH*, WT-pBAD, Δ*rpoH*-pBAD-*arpH*, and Δ*rpoH*-pBAD. We then used qRT-PCR to measure the mRNA levels of the invasion-associated gene *invF* and examined the invasion efficiency of these strains into HeLa cells. We concluded that the mRNA levels of *invF* and invasion efficiency of the WT-pBAD-*arpH* strain were lower than that of the WT-pBAD strain. However, when ArpH was overexpressed in the *rpoH* mutant, mRNA levels of *invF* and invasion into HeLa cells were not significantly different from the *rpoH* mutant alone (**Figure 8**). These results seem to indicate that the effect of ArpH on the invasiveness of *S.* Typhi may be due solely to its effects on *rpoH*. Additionally, the difference of twofold in the HeLa cell invasion assay may be statistically calculated to be significant, but in reality from a biological perspective it is probably almost insignificant. Therefore, the effect of ArpH on the invasiveness of *S*. Typhi needs further research to exclude experimental variation.

## Discussion

Many ncRNAs are involved in regulating the expression of genes involved in different biological processes through a variety of mechanisms (Loh et al., 2012; Chew et al., 2018). We applied deep sequencing analysis to discover ncRNAs in bacteria, including those involved in the invasion of epithelial cells by *Salmonella* (Dadzie et al., 2013, 2014; Gong et al., 2015; Zhang et al., 2015). Recently, novel ncRNAs associated with the response to stress were identified (Gong et al., 2011, 2015). By combining the findings of northern blotting and RACE analyses, we identified the *cis*-encoded ncRNA ArpH, which is complementary to *rpoH* mRNA. The full-length antisense RNA transcript was 3,508 nt from 411 nt upstream of the *yhhK* gene initiation codon to 238 nt upstream of the *rpoH* gene initiation codon on the complementary strand (**Figure 1**). No open reading frame region longer than 150 nt or Shine–Dalgarno sequence except for *yhhK* was identified in the entire sequence. Therefore, we believe that ArpH is a new ncRNA. It is worth mentioning that the *yhhK* gene might play an ancillary role in pantothenate biosynthesis in *S. enterica* (Stuecker et al., 2012). Because the *arpH*-encoding region overlaps the *yhhK* gene and the *arpH* might influence YhhK function. We constructed ArpH deletion mutant and overexpression strains away from *yhhK* coding region, which were estimated with qRT-PCR (Supplementary Figures S1, S2). In addition, the *livJ* gene is a pseudogene in *S.* Typhi.

We performed northern blotting and qRT-PCR to determine the expression of ArpH in *S.* Typhi. Peak expression of ArpH occurred in the late logarithmic phase (**Figure 2**). ArpH expression was reduced in acid stress conditions and increased in oxidative stress conditions (**Figure 3**). Based on these results, we investigated the effect of ArpH expression on bacterial growth. Growth curves of wild-type and ArpH overexpressing strains were similar during the lag and stationary phases. However, during the logarithmic phase, when the levels of endogenous ArpH transcripts were highest, the overexpression strain grew slightly more quickly than the wild-type strain (**Figure 5A**). Growth curves were also generated for WT-pBAD and WT-pBAD-*arpH* strains under different growth conditions. The growth curves of both strains were similar under osmotic stress. However, the growth of the WT-pBAD-*arpH* strain was enhanced under oxidative stress during the early logarithmic phase and overexpression of ArpH resulted in significant reduction of bacterial growth under acid stress during the late logarithmic phase (**Figures 5B–D**), indicating that the expression of ArpH enhances growth under oxidative stress conditions (Bang et al., 2005; Nuss et al., 2009; Remes et al., 2017). These findings are in accordance with the observation that, in general, ncRNAs are activated in response to different environmental conditions and that they function to help cells adapt to stress (Waters and Storz, 2009).

ncRNAs can regulate the translation of target genes by altering the stability of mRNAs (Ayupe and Reis, 2017). To clarify the effect of ArpH on its putative mRNA target *rpoH*, we investigated the expression levels of *rpoH* mRNA using qRT-PCR after overexpressing the partial *arpH* sequence from the arabinose-inducible recombinant plasmid WT-pBAD-*arpH*. *rpoH* mRNA levels increased gradually within 20 min of ArpH overexpression compared with WT-pBAD (**Figures 4A,B**). These results indicate that overexpression of ArpH positively regulates *rpoH* mRNA levels. To further determine the effect of ArpH overexpression on *rpoH* mRNA levels, we constructed RNase E and RNase III mutants. Expression levels of *rpoH* mRNA increased significantly in RNase III mutants (**Figure 4C**), whereas levels were relatively similar in RNase E mutants, indicating that RNase III may play a more significant role in the coupled degradation of the ArpH/RpoH duplex (Nicholson, 2014). Although some antisense RNAs inhibit target sense mRNA expression, many sense-antisense RNA pairs promote coordinated expression, suggesting that antisense RNAs may be involved in regulating the stability of *cis*-encoded mRNAs (Chinni et al., 2010; Lee and Groisman, 2010).

Microarray experiments showed that genes involved in SPI-1 and invasion, flagellar biosynthesis, and virulence were differentially expressed between WT-pBAD-*arpH* and WT-pBAD strains under oxidative stress conditions (see Supplementary Material). The superoxide dismutase gene *sodB* and the oxygen-regulated invasion gene *orgA* were upregulated in the WT-pBAD-*arpH* strain (**Figure 6**). The *orgA* gene is involved in promoting cellular invasion of the pathogen. Previously published work has indicated that the *prgH*, -*I*, -*J*, and -*K* genes are transcribed from a promoter distinct from that used by the gene immediately downstream, *orgA* (Klein et al., 2000). However, its exact role in virulence is still unclear mainly due to difficulties in understanding its complex regulation. Previous studies have been consistent regarding oxygen regulation of *orgA* (Russell et al., 2004). The reason may be that *orgA* is mainly regulated by oxidative stress. This suggests that ArpH may be involved in regulating the anti-oxygenation of *S.* Typhi under oxidative stress conditions (Glaeser et al., 2011). These findings are in agreement with the fact that the WT-pBAD-*arpH* strain grew more quickly than the WT-pBAD strain under oxidative stress conditions. Taken together, these data suggested that ArpH played a role in the antioxidant defenses of *S.* Typhi under oxidative stress conditions. These results are consistent with literature (Bang et al., 2005; Matsui et al., 2008). The microarray data revealed that the expression of flagellum-associated genes, such as *flgBCDEF*, *fliACJHLSZ*, and *flhDC*, were not significantly different in the WT-pBAD-*arpH* strain (see Supplementary Material). This indicates that ArpH does not participate in the regulation of *S*. Typhi motility. In addition, no significant changes were observed in the motility phenotypes of the WT-pBAD-*arpH* or WT-pBAD strains in motility agar (data not shown), which is in agreement with the microarray data.

A type III secretion system encoded by SPI-1 mediates the invasion of epithelial cells by *Salmonella* (Hensel, 2004). The deletion of ArpH increased bacterial invasion compared to wild-type cells (**Figure 7**). And the mRNA levels of *invF* and invasion efficiency of the WT-pBAD-*arpH* strain were lower than that of the WT-pBAD strain (**Figure 8**). This corresponds to the microarray results, which showed that invasion-associated genes, including *prgH* and *sipA*, were downregulated when ArpH was overexpressed (**Figure 6**). Our research is the first to demonstrate that ArpH is a player in host cell invasion by *S.* Typhi. It was previously reported that RpoH regulates virulence in many bacteria, including *Salmonella* (Bang et al., 2005; Delory et al., 2006; Grall et al., 2009; De la Cruz et al., 2016). Additionally, RpoH negatively regulates SPI-1 expression (Matsui et al., 2008). Due to the increase in expression of invasion-related genes, and in bacterial invasion observed in both *arpH* and *rpoH* deletion mutants, we hypothesize that ArpH may affect *Salmonella* invasiveness by acting on *rpoH* (**Figure 7**). When ArpH was overexpressed in *rpoH* mutants, the mRNA levels of *invF* and the invasion efficiency into HeLa epithelial cells were not significantly different from wild-type cells (**Figure 8**). These results seem to indicate that the effect of ArpH on the invasiveness of *S.* Typhi may be due solely to *rpoH*. We hypothesize that the mechanism by which ArpH affects *Salmonella* invasiveness is through the activation of *rpoH* expression (Bang et al., 2005; Matsui et al., 2008). However, while *rpoH* may have a small effect on the invasion phenotype, there are several confusing details about how *arpH* alters transcription of invasion genes, therefore, further detailed research is needed.

## Conclusion

The full-length *arpH* sequence was found to be 3,508 nt located 411 nt upstream of the *yhhK* initiation codon and 238 nt upstream of the *rpoH* initiation codon on the complementary strand. ArpH is likely to affect the expression of *rpoH* in *cis* at the transcriptional level.

## Author Contributions

CX and XH conceived and designed the experiments. CX, XL, XZ, and JL performed the experiments. CX, XL, and XH analyzed the data. CX, SX, and XL contributed reagents, materials, and analysis tools. CX and XH wrote the manuscript.

## Conflict of Interest Statement

The authors declare that the research was conducted in the absence of any commercial or financial relationships that could be construed as a potential conflict of interest.
