# Supplementary material for: Identification and Characterization of a Cis Antisense RNA of the rpoH Gene of Salmonella enterica Serovar Typhi
Source: Front Microbiol. 2018 May 15;9:978. doi: 10.3389/fmicb.2018.00978 (PMC5963218; doi:10.3389/fmicb.2018.00978)
Supplement: Supplementary file 1 [file Data_Sheet_1.DOC]

Gene expression changes in WT-pBAD-*arpH* strain compared to the WT-pBAD strain of *S*. Typhi under oxidative stress (Log_2_ R^a^ = Log_2_ (WT-pBAD-*arpH* / WT-pBAD) ^a^

| Functional kinds | Product function | Genes | Log_2_ R^a^ |
| --- | --- | --- | --- |
| **Downregulated** |  | | |
| Invasion proteins | cell invasion protein | *sigD* | -3.15 |
|  | cell invasion protein | *sigE* | -2.46 |
|  | possible AraC-family transcriptional regulator | *sprB* | -3.01 |
|  | possible AraC-family transcriptional regulator | *sprA* | -2.59 |
|  | pathogenicity 1 island effector protein | *prgK* | -3.25 |
|  | pathogenicity 1 island effector protein | *prgI* | -3.61 |
|  | pathogenicity 1 island effector protein | *prgH* | -3.39 |
|  | invasion protein regulator | *iagA* | -2.52 |
|  | cell invasion protein | *iagB* | -2.48 |
|  | acyl carrier protein | *sipF* | -2.46 |
|  | pathogenicity island 1 effector protein | *sipA* | -3.32 |
|  | pathogenicity island 1 effector protein | *sipD* | -3.05 |
|  | pathogenicity island 1 effector protein | *sipC* | -2.25 |
|  | unknown function | *spaT* | -1.36 |
|  | type III secretion protein | *spaS* | -2.09 |
|  | virulence-associated secretory protein | *spaR* | -2.43 |
|  | virulence-associated secretory protein | *spaQ* | -1.89 |
|  | virulence-associated secretory protein | *spaP* | -3.78 |
|  | type III secretion protein | *spaO* | -2.96 |
|  | antigen presentation protein SpaN | *spaN* | -3.90 |
|  | virulence-associated secretory protein | *spaM* | -3.47 |
|  | type III secretion system ATPase | *spaI* | -3.06 |
|  | virulence-associated secretory protein | *spaK* | -3.71 |
|  | possible virulence-associated secretory protein | *invA* | -3.06 |
|  | cell invasion protein | *invE* | -3.75 |
|  | virulence-associated secretory protein | *invG* | -2.93 |
|  | possible AraC-family regulatory protein | *invF* | -2.72 |
|  | cell adherance/invasion protein | *invH* | -2.74 |
|  | putative virulence effector protein | *srfA* | -3.05 |
|  | putative virulence effector protein | *srfB* | -3.41 |
|  | putative virulence effector protein | *srfC* | -3.24 |
|  | invasion-associated secreted protein | *sopE* | -2.65 |
| Vi -locus | Vi polysaccharide biosynthesis protein | *tviA* | -3.52 |
|  | Vi polysaccharide biosynthesis protein, epimerase | *tviC* | -2.41 |
|  | Vi polysaccharide biosynthesis protein | *tviD* | -2.85 |
|  | Vi polysaccharide biosynthesis protein | *tviE* | -3.91 |
| Hypothetical proteins | hypothetical protein t0349 | - | -1.53 |
|  | hypothetical protein t0552 | - | -1.72 |
|  | hypothetical protein t0804 | - | -1.37 |
|  | hypothetical protein t0892 | - | -1.90 |
|  | hypothetical protein t0893 | *yodD* | -1.27 |
|  | hypothetical protein t0910 | - | -1.57 |
|  | hypothetical protein t0911 | *yedF* | -1.29 |
|  | hypothetical protein t0927 | *yecF* | -1.55 |
|  | hypothetical protein t0950 | *yecG* | -1.69 |
|  | hypothetical protein t0996 | *yebF* | -1.27 |
|  | hypothetical protein t0997 | *yebE* | -1.57 |
|  | hypothetical protein t1080 | - | -1.38 |
|  | hypothetical protein t1145 | - | -1.59 |
|  | hypothetical protein t1153 | - | -1.28 |
|  | hypothetical protein t1179 | - | -1.83 |
|  | hypothetical protein t1187 | - | -1.93 |
|  | hypothetical protein t1238 | - | -1.48 |
|  | hypothetical protein t1240 | - | -1.62 |
|  | hypothetical protein t1434 | - | -1.42 |
|  | hypothetical protein t1479 | - | -1.34 |
|  | hypothetical protein t1510 | *ugtL* | -1.04 |
|  | hypothetical protein t1640 | - | -1.63 |
|  | hypothetical protein t1641 | - | -1.57 |
|  | hypothetical protein t1642 | - | -2.09 |
|  | hypothetical protein t1643 | - | -1.52 |
|  | hypothetical protein t1678 | - | -1.13 |
|  | hypothetical protein t1773 | - | -1.10 |
|  | hypothetical protein t1774 | - | -1.62 |
|  | hypothetical protein t1800 | - | -2.47 |
|  | hypothetical protein t1802 | - | -1.71 |
|  | hypothetical protein t1834 | - | -1.28 |
|  | hypothetical protein t2053 | *ybiP* | -1.26 |
|  | hypothetical protein t2075 | *ybhP* | -1.27 |
|  | hypothetical protein t2475 | *yaiA* | -1.04 |
|  | hypothetical protein t2698 | - | -1.57 |
|  | hypothetical protein t2782 | - | -1.48 |
|  | hypothetical protein t2811 | - | -1.52 |
|  | hypothetical protein t3059 | - | -1.53 |
|  | hypothetical protein t3075 | - | -1.61 |
|  | hypothetical protein t3076 | - | -1.94 |
|  | hypothetical protein t3148 | - | -1.33 |
|  | hypothetical protein t3150 | - | -1.73 |
|  | hypothetical protein t3153 | - | -1.11 |
|  | hypothetical protein t3189 | - | -1.68 |
|  | hypothetical protein t3292 | - | -1.57 |
|  | hypothetical protein t3349 | *yigA* | -1.58 |
|  | hypothetical protein t3539 | - | -1.56 |
|  | hypothetical protein t3619 | - | -2.83 |
|  | hypothetical protein t3906 | *yhjH* | -2.28 |
|  | hypothetical protein t3907 | - | -1.67 |
|  | hypothetical protein t3944 | *yhhP* | -1.27 |
|  | hypothetical protein t4034 | *yhfG* | -1.55 |
|  | hypothetical protein t4148 | - | -1.57 |
|  | hypothetical protein t4195 | *phnB* | -1.15 |
|  | hypothetical protein t4459 | *ytfK* | -1.72 |
|  | hypothetical protein t4605 | - | -1.28 |
| Metabolism-associated proteins | sugar fermentation stimulation protein | *sfsA* | -1.25 |
|  | Succinyl arginine double enzyme hydrolysis | *astB* | -3.05 |
|  | succinate dehydrogenase cytochrome b556 small membrane subunit | *sdhD* | -2.55 |
|  | succinate dehydrogenase cytochrome b556 large membrane subunit | *sdhC* | -2.83 |
|  | transketolase | *tktB* | -1.53 |
|  | transketolase | *talA* | -1.83 |
|  | glucose-specific PTS system enzyme IIA component | *crr* | -1.58 |
|  | putative decarboxylase | - | -1.83 |
|  | lipid A biosynthesis lauroyl acyltransferase | - | -1.03 |
|  | fructose-bisphosphate aldolase | *fbaB* | -1.37 |
|  | UTP-glucose-1-phosphate uridylyltransferase | *galF* | -1.84 |
|  | cytoplasmic alpha-amylase | *amyA* | -1.57 |
|  | D-cysteine desulfhydrase | - | -1.82 |
|  | putative lipoprotein | - | -1.57 |
|  | trehalose phosphatase | *otsB* | -1.83 |
|  | trehalose-6-phosphate synthase | *otsA* | -1.98 |
|  | putative hydrolase | *yjcS* | -1.43 |
|  | osmotically inducible lipoprotein E precursor | *osmE* | -1.58 |
|  | catalase HPII | *katE* | -1.21 |
|  | selenocysteine lyase | - | -1.03 |
|  | cysteine desufuration protein SufE | - | -1.46 |
|  | pyruvate kinase | *pykF* | -1.28 |
|  | copper-zinc superoxide dismutase | *sodC* | -1.57 |
|  | putative glycogen debranching protein | *glgX* | -2.58 |
|  | putative hydrolase | - | -2.82 |
|  | putative hydrolase | - | -2.02 |
|  | respiratory nitrate reductase 2 alpha chain | *narZ* | -1.93 |
|  | respiratory nitrate reductase 2 alpha chain | *narY* | -1.57 |
|  | putative lipoprotein | - | -1.83 |
|  | putative NADP-dependent oxidoreductase | - | -1.03 |
|  | putative permease transmembrane component | *yehY* | -1.82 |
|  | polysaccharide chain length regulator | *cld* | -1.52 |
|  | putative aldehyde dehydrogenase | - | -1.05 |
|  | probable pyruvate-flavodoxin oxidoreductase | - | -1.81 |
|  | respiratory nitrate reductase 1 delta chain | *narJ* | -1.72 |
|  | respiratory nitrate reductase 1 beta chain | *narH* | -1.79 |
|  | glutaredoxin 2 | *grxB* | -1.59 |
|  | exonuclease | *exo* | -1.83 |
|  | formate acetyltransferase 1 | *pflB* | -1.82 |
|  | pyruvate dehydrogenase | *poxB* | -1.52 |
|  | putative oxidoreductase | *yliI* | -1.49 |
|  | glutamine ABC transporter periplasmic-binding protein | *glnH* | -1.48 |
|  | putative phospholipase | *ybhO* | -1.72 |
|  | deoxyribodipyrimidine photolyase | *phrB* | -1.28 |
|  | conserved hypothetical lipoprotein | - | -1.60 |
|  | putative oxidoreductase | - | -1.94 |
|  | probable terminal oxidase subunit I | - | -2.01 |
|  | carbon storage regulator | *csrA* | -1.14 |
|  | tyrosine phosphatase | *stpA* | -1.04 |
|  | possible lipoprotein | - | -1.36 |
|  | possible transferase | - | -1.53 |
|  | oxidoreductase | - | -2.83 |
|  | threonine dehydratase | *tdcB* | -1.80 |
|  | glycerol uptake facilitator protein | *glpF* | -2.25 |
|  | glycerol kinase | *glpK* | -2.58 |
|  | fructose 1,6-bisphosphatase II | *glpX* | -1.81 |
|  | putative glycosyl hydrolase | - | -1.26 |
|  | putative glutathione transferase | - | -1.05 |
|  | putative sugar kinase | - | -1.23 |
|  | aldehyde dehydrogenase B | *aldB* | -1.43 |
|  | cytoplasmic trehalase | *treF* | -1.05 |
|  | methyl-accepting chemotaxis citrate transducer | - | -2.03 |
|  | sn-Glycerol-3-phosphate transport system permease protein | *ugpE* | -1.01 |
|  | aerobic glycerol-3-phosphate dehydrogenase | *glpD* | -2.84 |
|  | malate synthase | *aceB* | -1.32 |
|  | isocitrate lyase | *aceA* | -1.24 |
|  | putative glutathione S transferase | - | -1.62 |
|  | alpha-galactosidase | *melA* | -1.83 |
|  | putative lipoprotein | *bcl* | -1.47 |
|  | Acetyl ornithine aminotransferase | *argD* | -2.72 |
| Other functional proteins | ATP-dependent RNA helicase | *srmB* | -1.13 |
|  | putative receptor/regulator protein | - | -2.31 |
|  | putative periplasmic protein | *yehZ* | -2.56 |
|  | ABC transporter ATP-binding protein | *yehX* | -1.35 |
|  | putative transcriptional regulator | *-* | -1.52 |
|  | DsrB protein | *dsrB* | -1.03 |
|  | putative ABC transport ATP-binding protein | - | -1.07 |
|  | cell-division regulatory protein | *sdiA* | -2.06 |
|  | putative MutT-family protein | - | -1.46 |
|  | putative outer membrane protein | - | -1.83 |
|  | putative ABC transport ATP-binding subunit | - | -1.56 |
|  | putative ABC transporter ATP/GTP-binding protein | - | -1.66 |
|  | putative ABC transporter membrane protein | - | -1.56 |
|  | putative ABC transporter periplasmic binding protein | - | -1.83 |
|  | putative periplasmic protein | - | -1.37 |
|  | membrane transport protein | - | -1.94 |
|  | putative secreted protein | - | -1.47 |
|  | haemolysin HlyE | *hlyE* | -1.58 |
|  | osmotically inducible protein C | *osmC* | -1.83 |
|  | 30S ribosomal protein S22 | *rpsV* | -1.95 |
|  | putative regulatory protein | - | -1.76 |
|  | putative chemo-receptor protein | - | -1.84 |
|  | osmotically inducible lipoprotein B precursor | *osmC* | -1.57 |
|  | TonB protein | *tonB* | -2.37 |
|  | cation transport regulator ChaB | *chaB* | -1.08 |
|  | acidic protein MsyB | *msyB* | -3.01 |
|  | PhoH protein | *phoH* | -1.55 |
|  | TrpR binding protein WrbA | *wrbA* | -1.51 |
|  | conserved hypothetical bacteriophage protein | - | -1.71 |
|  | DNA replication protein DnaC | - | -1.83 |
|  | conserved hypothetical bacteriophage protein | - | -1.53 |
|  | DNA protection during starvation conditions | *dps* | -1.96 |
|  | probable secreted protein | - | -2.53 |
|  | lysR-family transcriptional regulator | *ybeF* | -1.83 |
|  | phosphate starvation-inducible protein PsiF | *psiF* | -1.39 |
|  | probable secreted protein | - | -1.41 |
|  | putative transcriptional regulator | - | -1.50 |
|  | chaperone | *sicP* | -1.97 |
|  | possible LysR-family transcriptional regulator | - | -1.24 |
|  | aerotaxis receptor protein | *air* | -1.08 |
|  | extracytoplasmic stress protein for protein-mediated toxicities | *cpxP* | -1.82 |
|  | putative secreted protein | - | -1.92 |
|  | putative DNA-binding protein | - | -1.03 |
|  | cell filamentation protein Fic | *fic* | -1.57 |
|  | bacterioferritin | *bfr* | -1.84 |
|  | ProP protein | *proP* | -1.58 |
|  | entericidin B precursor | *ecnB* | -1.79 |
|  | transcriptional regulatory protein | *ecnR* | -1.22 |
|  | SugE protein | *sugE* | -1.84 |
|  | regulatory protein | - | -1.94 |
|  | Putative periplasmic protein | *osmY* | -1.53 |
| **Upregulated** |  | | |
| Regulators | molecular chaperone DnaK | *dnaK* | 1.48 |
|  | co-chaperonin GroES | *groES* | 1.92 |
|  | chaperonin GroEL | *groEL* | 1.03 |
| Ribosomal proteins | 30S ribosomal protein S10 | *rpsJ* | 1.57 |
|  | 50S ribosomal protein L3 | *rplC* | 1.39 |
|  | 50S ribosomal protein L4 | *rplD* | 1.92 |
|  | 50S ribosomal protein L23 | *rplW* | 1.03 |
|  | 50S ribosomal protein L2 | *rplB* | 1.26 |
|  | 30S ribosomal protein S19 | *rpsS* | 1.76 |
|  | 50S ribosomal protein L22 | *rplV* | 1.93 |
|  | 30S ribosomal protein S3 | *rpsC* | 1.03 |
|  | 50S ribosomal protein L16 | *rplP* | 1.83 |
|  | 50S ribosomal protein L29 | *rpmC* | 1.29 |
|  | 30S ribosomal protein S17 | *rpsQ* | 1.28 |
|  | 30S ribosomal protein S14 | *rpsN* | 1.77 |
|  | 30S ribosomal protein S8 | *rpsH* | 1.23 |
|  | 50S ribosomal protein L6 | *rplF* | 1.93 |
|  | 50S ribosomal protein L18 | *rplR* | 1.36 |
|  | 30S ribosomal protein S5 | *rpsE* | 1.63 |
|  | 50S ribosomal protein L30 | *rpmD* | 1.26 |
|  | 50S ribosomal protein L15 | *rplO* | 1.82 |
|  | 30S ribosomal protein S11 | *rpsK* | 1.63 |
| Metabolism-associated proteins | preprotein translocase SecY | *prlA* | 1.23 |
|  | DNA-directed RNA polymerase alpha subunit | *rpoA* | 1.64 |
|  | aconitate hydratase | *acnB* | 1.85 |
|  | 2-keto-3-deoxygluconate permease | *kdgT* | 1.73 |
|  | putative exported amidase | *bax* | 1.01 |
|  | putative sugar phosphotransferase component II A | - | 1.02 |
|  | putative sugar phosphotransferase component II B | - | 1.55 |
|  | ascorbate-specific PTS system enzyme IIC | *ulaA* | 2.46 |
|  | putative transketolase C-terminal section | - | 2.83 |
|  | NADH dehydrogenase operon transcriptional regulator | *lrhA* | 1.52 |
|  | ferredoxin-type protein NapF | *napF* | 1.15 |
|  | D-galactose-binding periplasmic protein precursor | *mglB* | 1.38 |
|  | cytidine deaminase | *cdd* | 1.49 |
|  | alanine racemase | *alr* | 1.83 |
|  | uptake hydrogenase small subunit | *hyaA* | 1.04 |
|  | oxidoreductase | - | 1.69 |
|  | histidine ammonia-lyase | *hutH* | 1.59 |
|  | UDP-glucose 4-epimerase | *galE* | 1.93 |
|  | succinate dehydrogenase catalytic subunit | *sdhB* | 1.58 |
|  | succinate dehydrogenase catalytic subunit | *sdhA* | 2.02 |
|  | putative two-component system sensor kinase | *tctE* | 1.09 |
|  | glycine betaine/L-proline transport system permease protein P | *proW* | 1.44 |
|  | nucleoside permease | *nupG* | 1.83 |
|  | mannonate dehydratase | *uxuA* | 1.93 |
|  | D-mannonate oxidoreductase | *uxuB* | 1.65 |
|  | putative sialic acid transporter | *nanT* | 1.90 |
|  | N-acetylneuraminate lyase | *nanA* | 1.51 |
|  | malate dehydrogenase | *mdh* | 1.35 |
|  | acetyl-CoA acetyltransferase | *fadA* | 1.83 |
|  | uridine phosphorylase | *udp* | 1.47 |
|  | ketol-acid reductoisomerase | *ilvC* | 1.94 |
|  | terminase, ATPase subunit | - | 1.37 |
|  | Magnesium transport ATPase, P-type 2 | *mgtB* | 1.15 |
|  | formamidopyrimidine-DNA glycosylase | *mutM* | 1.40 |
|  | putative RNA methyltransferase | *yibK* | 1.47 |
|  | putative L-lactate dehydrogenase | *lctD* | 2.37 |
| Other-functional proteins | oxygen-regulated invasion protein | *orgA* | 2.15 |
|  | superoxide dismutase | *sodB* | 3.86 |
|  | outer membrane protein F precursor | *ompF* | 1.93 |
|  | outer membrane protein X | *ompX* | 1.71 |
|  | putative molybdenum transport protein ModE | *modE* | 1.68 |
|  | heat shock protein 90 | *htpG* | 1.46 |
|  | peptide chain release factor 2 | *prfH* | 1.65 |
|  | putative GAB DTP gene cluster repressor | *ygaF* | 1.56 |
|  | chloramphenicol-sensitive protein RarD | - | 1.53 |
|  | putative phage tail protein | - | 1.93 |
|  | cytochrome c-type biogenesis protein F2 | *ccmF* | 1.38 |
|  | heat shock protein A | *hslT* | 2.06 |
|  | heat shock protein B | *hslS* | 2.12 |
|  | elongation factor EF-2 | *fusA* | 1.57 |
| Hypothetical proteins | hypothetical protein t2145 | - | 1.04 |
|  | hypothetical protein t2684 | - | 1.06 |
|  | hypothetical protein t2943 | - | 1.08 |
|  | hypothetical protein t3052 | - | 1.34 |
|  | hypothetical protein t3578 | - | 1.38 |
|  | hypothetical protein t3579 | - | 1.83 |
|  | hypothetical protein t3867 | - | 1.57 |
| Flagella and chemotaxis proteins | flagellar biosynthesis protein | *fliQ* | 0.23 |
|  | flagellar biosynthesis protein | *flip* | 0.41 |
|  | flagellar protein FliO | *fliO* | 0.32 |
|  | flagellar motor switch protein | *fliN* | 0.61 |
|  | flagellar motor switch protein | *fliM* | 0.03 |
|  | flagellar biosynthesis protein | *fliL* | 0.05 |
|  | flagellar protein | *fliJ* | 0.04 |
|  | flagellar assembly protein | *fliH* | 0.09 |
|  | flagellar motor protein | *fliG* | 0.15 |
|  | flagellar M-ring protein | *fliF* | 0.51 |
|  | flagellar M-ring protein | *fliE* | 0.69 |
|  | flagellar protein FliS | *fliS* | 0.06 |
|  | flagellar hook-associated protein | *fliD* | 0.58 |
|  | flagellar biosynthesis protein | *fliC* | 0.06 |
|  | flagellar biosynthesis sigma factor FliA | *fliA* | 0.01 |
|  | FliZ protein | *fliZ* | 0.04 |
|  | cystine-binding periplasmic protein precursor | *fliY* | 0.45 |
|  | cystine-binding periplasmic protein precursor | *flhD* | 0.04 |
|  | flagellar transcriptional activator | *flhC* | 0.07 |
|  | flagellar motor protein | *motA* | 0.46 |
|  | chemotaxis protein CheA | *cheA* | 0.64 |
|  | purine binding chemotaxis protein | *cheW* | 0.27 |
|  | methyl-accepting chemotaxis protein II | *tar* | 0.72 |
|  | chemotaxis protein methyltransferase | *cheR* | 0.47 |
|  | chemotaxis-specific methylesterase | *cheB* | 0.08 |
|  | chemotaxis protein CheY | *cheY* | 0.74 |
|  | chemotaxis protein CheZ | *cheZ* | 0.61 |
|  | flagellar biosynthesis protein | *flhB* | 0.74 |
|  | flagellar biosynthesis protein | *flhA* | 0.25 |
|  | methyl-accepting chemotaxis protein II | *cheM* | 0.36 |
|  | flagellar hook-associated protein | *flgL* | 0.62 |
|  | flagellar hook-associated protein | *flgK* | 0.14 |
|  | flagellar biosynthesis protein | *flgJ* | 0.75 |
|  | flagellar P-ring protein precursor | *flgI* | 0.48 |
|  | flagellar L-ring protein precursor | *flgH* | 0.37 |
|  | putative flagellar basal-body rod protein FlgF | *flgF* | 0.02 |
|  | flagellar hook protein | *flgE* | 0.06 |
|  | flagellar basal body rod modification protein | *flgD* | 0.03 |
|  | flagellar basal body rod protein | *flgC* | 0.08 |
|  | flagellar basal body rod protein | *flgB* | 0.02 |
|  | flagellar basal body P-ring biosynthesis protein | *flgA* | 0.46 |
|  | negative regulator of flagellin synthesis | *flgM* | 0.74 |
|  | flagella synthesis protein FlgN | *flgN* | 0.52 |
