# Supplementary material for: Identification and Characterization of a Cis Antisense RNA of the rpoH Gene of Salmonella enterica Serovar Typhi
Source: Front Microbiol. 2018 May 15;9:978. doi: 10.3389/fmicb.2018.00978 (PMC5963218; doi:10.3389/fmicb.2018.00978)
Supplement: Supplementary file 2 [file Data_Sheet_2.DOCX]

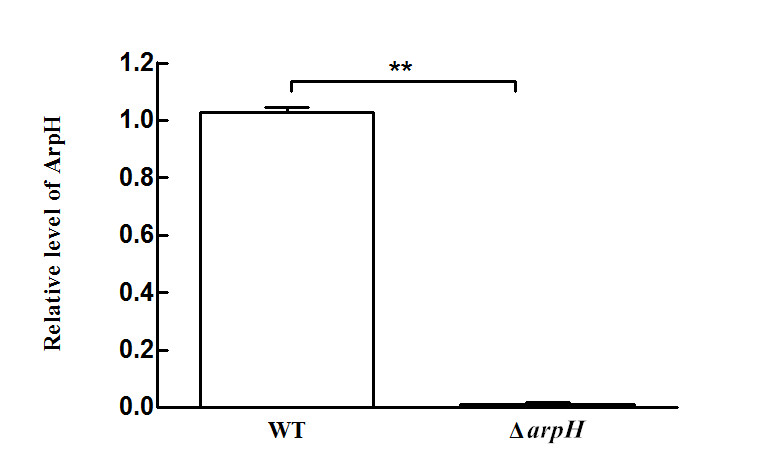


**Figure S1. Expression of ArpH in WT and** **Δ*arpH* strains.**

Expression of ArpH was determined by qRT-PCR. RNA was extracted from WT and Δ*arpH* strain grown in LB to OD_600_ 0.8. Levels of 5S rRNA were the internal reference. ** *P* < 0.01 compared with WT control group.


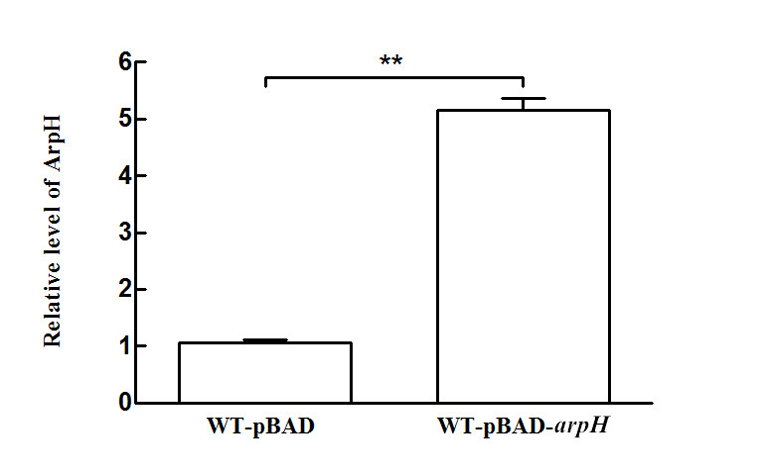


**Figure S2. Expression of ArpH in** **WT-pBAD and** **WT-pBAD-*arpH* strains.**

Expression of ArpH was determined by qRT-PCR. RNA was extracted from WT-pBAD strain and WT-pBAD-*arpH* strain, grown in LB to OD_600_ 0.4 and induced with the addition of 0.2% L-arabinose. Levels of 5S rRNA were the internal reference. ** *P* < 0.01 compared with WT-pBAD control group.
